# Supplementary material for: Microbe-Based Sensor for Long-Term Detection of Urine Glucose
Source: Sensors (Basel). 2022 Jul 17;22(14):5340. doi: 10.3390/s22145340 (PMC9320042; doi:10.3390/s22145340)
Supplement: Supplementary file 1 [file sensors-22-05340-s001.zip › sensors-1803246-supplementary.pdf]

-Supplementary material-

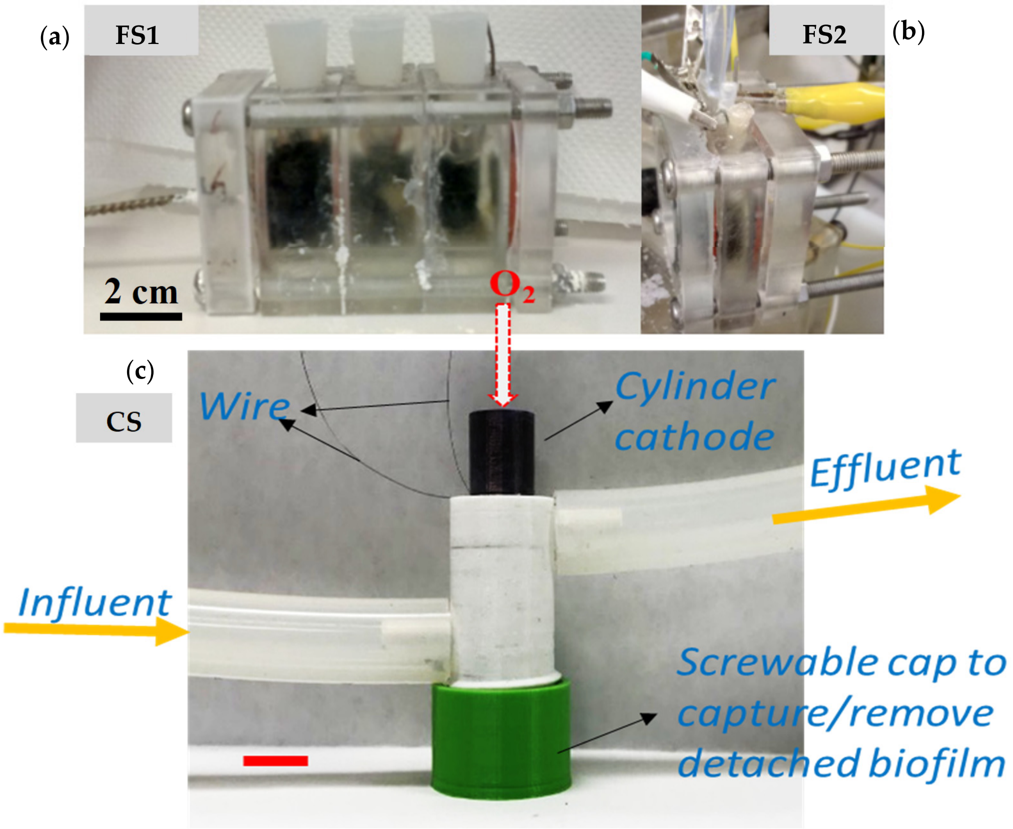

Figure S1. Pictures of (a) FS1, (b) FS2 and (c) CS used in this study. (a) and (b) share the same scale bar. The red scale bar in (c) indicates 1 cm.

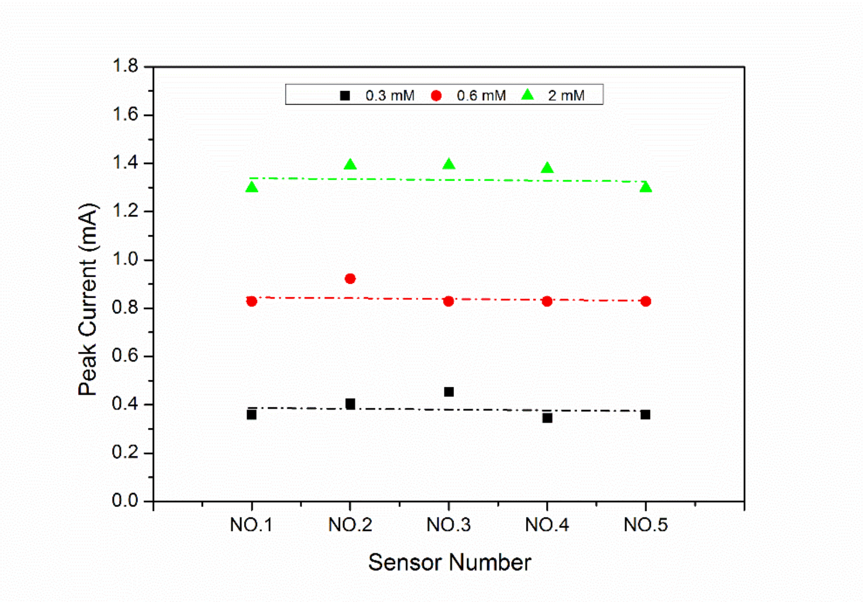

Figure S2. Reproducibility on the peak current response for different concentrations of glucose solutions (FS1)

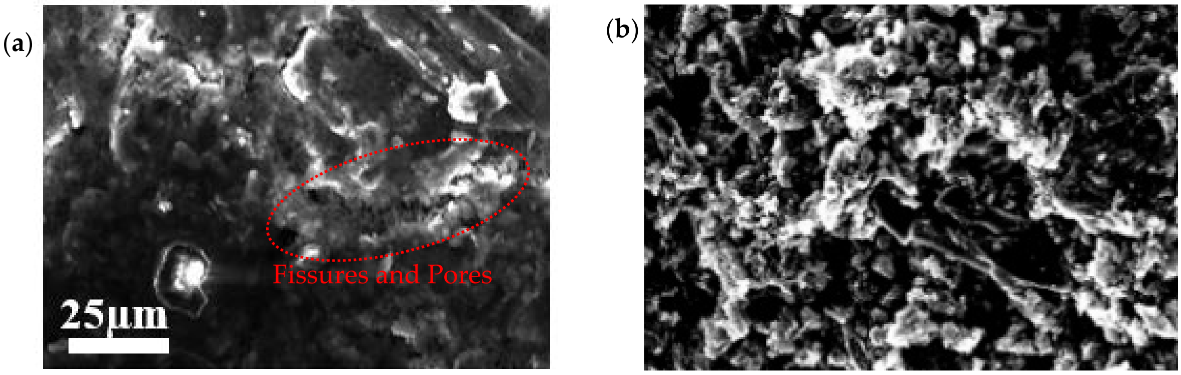

Figure S3. SEM images of the (**a**) new and (**b**) used CS cathode, respectively.

Two images share the same scale bar at figure S3a.

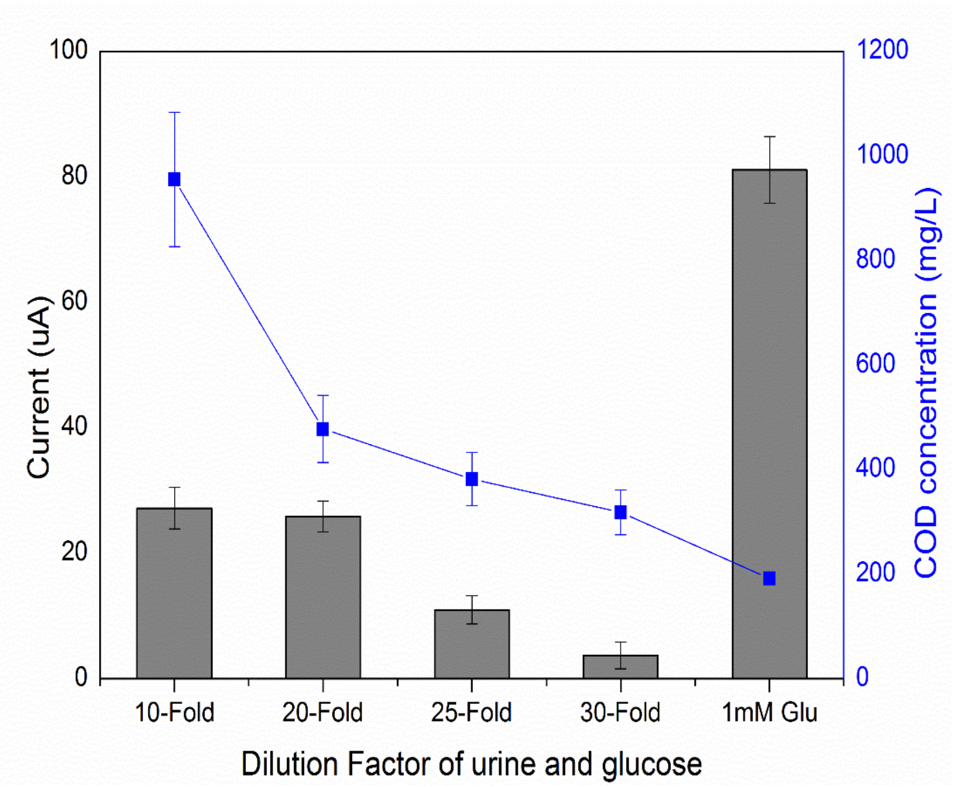

Figure S4. Current of CS in the presence of 1mM glucose solution, and normal urine at different dilution factors. (The urine sample was collected from 4 healthy individuals and mixed together for testing. The glucose concentration of this mixed urine was tested by a commercial analyser and was found to be was less than 0.2 mM). Dilution factors of 10-20 times yielded in relatively high currents (27.2 ± 3.3 μA in 15 mins). When the dilution factor increased to 25 times, the current obtained from the diabetes negative urine samples was negligible,only accounting to 13.6% of detection limit of Cyl. Although the COD concentration of urine was much higher than 1mM glucose, there was no stable current generated by the Cyl when the urine was diluted by 30-fold. Hence, the background interference of other organics in urine could be avoided when the dilution factor is greater than 20-30 times.

Table S1 The composition of mineral and vitamin.

Minerals		Vitamin	
Name	Concentration (g/L)	Name	Concentration (mg/L)
$ZnCl_2$	0.13	Vitamin B1	5
$MgSO_4{\cdot}7H_2O$	6.15	Vitamin B2	5
$MnSO_4{\cdot}H_2O$	0.5	Vitamin B3	5
NaCl	1	Vitamin B5	5
$Na_2MoO_4{\cdot}2H_2O$	0.054	Vitamin B6	10
H_3BO_3	0.01	Vitamin B9	2
$FeSO_4{\cdot}7H_2O$	0.1	Vitamin B12	0.1
$CuSO_4{\cdot}5H_2O$	0.01	Biotin	2
$CoCl_2{\cdot}6H_2O$	0.1	4-aminobenzoic acid	5
$CaCl_2{\cdot}2H_2O$	0.1	Lipoic acid	5
Nitrilotriacetic acid (NTA)	1.5		
$AlK(SO_4)_2{\cdot}12H_2O$	0.01		

Table S2 Material and cost analysis of CS

Cyl Component	Price	Material for 1 Cyl	Cost for 1 Cyl	Total cost of 1 Cyl	Cost of each test [b]
Anode	\$ 0.27/each	1	\$ 0.27		
Cathode [a]	\$ 15/m^2	5.5 cm^2	\$ 0.008		
Separator-demine fabric	\$ 13.8/m2	5.5 cm2	\$ 0.008	\$ 0.47	\$ 0.00312
Chamber	\$ 0.08/each	2	\$ 0.172		
Ti wire	\$ 0.1/m	10cm	\$ 0.01		

a: Obtained from [1], other material price obtained from Alibaba.com b: Assuming 5 months stable operation, test frequency is 1 time/day.

1. Yang, W.; He, W.; Zhang, F.; Hickner, M. A.; Logan, B. E., Single-Step Fabrication Using a Phase Inversion Method of Poly(vinylidene fluoride) (PVDF) Activated Carbon Air Cathodes for Microbial Fuel Cells. *Environmental Science & Technology Letters* **2014,** 1, (10), 416-420.
